# Rapidly progressive respiratory failure after helminth larvae ingestion

**DOI:** 10.1186/s12890-021-01788-w

**Published:** 2021-12-20

**Authors:** Anahit A. Zeynalyan, Balaji Kolasani, Chetan Naik, Christopher J. G. Sigakis, Leann Silhan, Susan K. Mathai

**Affiliations:** 1 Department of Internal Medicine, Baylor University Medical Center, 3500 Gaston Ave, Dallas, Texas 75246 USA; 2 Department of Internal Medicine - Pulmonary Research, UT Southwestern Medical Center, Dallas, Texas USA; 3 Center for Advanced Heart and Lung Disease, Baylor University Medical Center, Dallas, Texas USA; 4 Department of Radiology, Cleveland Clinic Foundation, Cleveland, Ohio USA; 5 Leann Silhan, MD PLLC, Dallas, Texas USA

**Keywords:** Löffler’s syndrome, Helminth therapy, Pulmonary arterial hypertension, Systemic sclerosis, Scleroderma, Eosinophilic lung disease

## Abstract

**Background:**

Self-administration of helminths has gained attention among patients as a potential but unproven therapy for autoimmune disease. We present a case of rapidly progressive respiratory failure in a patient with systemic sclerosis (SSc) and pulmonary arterial hypertension (PAH) as a result of self-administration of parasitic organisms.

**Case:**

A 45-year-old woman with a history of interstitial lung disease and PAH due to limited cutaneous SSc presented to pulmonary clinic with worsening dyspnea, cough, and new onset hypoxemia. Three months prior to presentation she started oral helminth therapy with *Necator americanus* as an alternative treatment for SSc. Laboratory evaluation revelaed eosinophilia and elevated IgE levels. IgG antibodies to Strongyloides were detected. High resolution computed tomography of the chest revealed progressive ILD and new diffuse ground glass opacities. Transthoracic echocardiogram and right heart catheterization illustrated worsening PAH and right heart failure. The patient was admitted to the hospital and emergently evaluated for lung transplantation but was not a candidate for transplantation due to comorbidities. Despite aggressive treatment for PAH and right heart failure, her respiratory status deteriorated, and the patient transitioned to comfort-focused care.

**Conclusion:**

Although ingestion of helminths poses a risk of infection, helminth therapy has been investigated as a potential treatment for autoimmune diseases. In this case, self-prescribed helminth ingestion precipitated fatal acute worsening of lung inflammation, hypoxemia, and right heart dysfunction, highlighting the risk of experimental helminth therapy in patients, especially those with underlying respiratory disease.

## Background

Therapeutic ingestion of parasitic organisms has gained interest among patients with autoimmune disease as a potential immune-modulating therapy, though rigorous clinical studies of this approach are lacking. Parasitic infections have known pulmonary complications such as Löffler’s syndrome, an acute eosinophilic pneumonitis precipitated by parasitic infection. It is characterized by self-limiting pulmonary symptoms, transient radiographic changes in lung fields, and peripheral blood eosinophilia. In rare occasions, large helminthic burden or underlying pulmonary disease can predispose to prolonged eosinophilic pulmonary inflammation [1]. To our knowledge, there are no reported cases of this syndrome occurring due to an intentional ingestion of parasitic organisms. Here, we present a case of rapidly progressive respiratory decline and right heart failure in a patient who self-administered parasitic organisms in an effort to treat her autoimmune disease.

## Case presentation

**Fig. 1:**
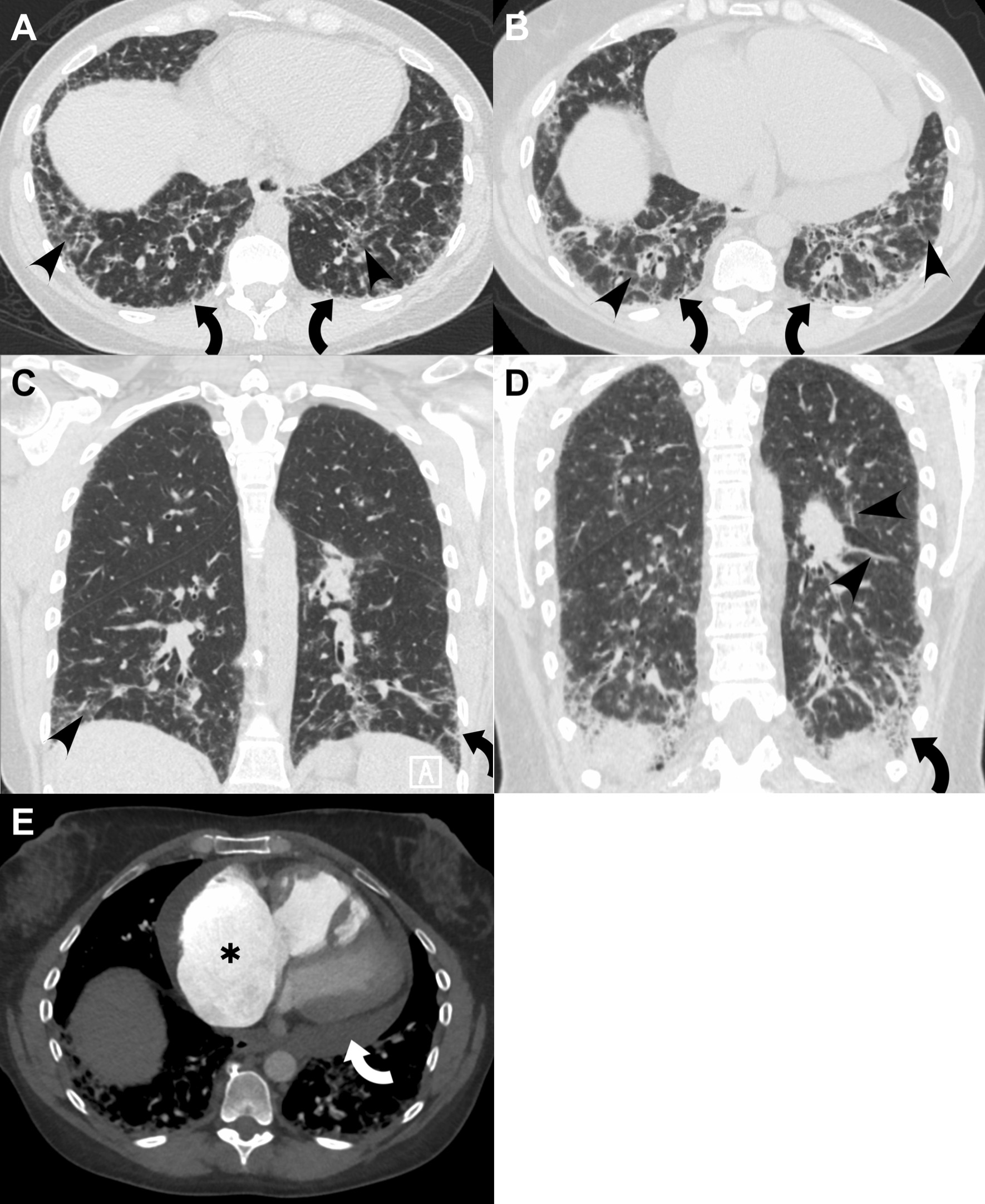
**A** Axial image from high resolution CT (HRCT) scan of the chest 4 years prior to decompensation, showing subpleural reticulation (curved arrows) and traction bronchiolectasis as well as ground glass opacities (arrowheads). Heart size was at that time normal. **B** Axial image from HRCT scan performed after patient's respiratory decline, showing increased ground glass abnormality (arrowheads) as well as progressive fibrosis denoted by increased subpleural reticular abnormality and traction bronchiolectasis (curved arrows). **C** Coronal image from prior HRCT scan of chest illustrating reticular abnormality (curved arrows) and ground glass opacity (arrowhead). **D** Coronal image from HRCT performed after patient's clinical decline, indicating increased diffusing ground glass opacities, worsened fibrotic change (curved arrow), and irregular linear bands not conforming to normal pulmonary vasculature, possibly reflecting additional helminth infiltration of the lung parenchyma. **E** Post-contrast CT chest performed during the patient's hospitalization with enlarged right heart chambers (marked right atrial dilatation, starred) and flattened interventricular septum indicating right heart strain. A moderate pericardial effusion (curved arrow) is also present

A 45-year-old woman presented to pulmonary clinic for evaluation of worsening dyspnea, cough, and hypoxemia. Her medical history was significant for limited cutaneous systemic sclerosis (lcSSc), pulmonary arterial hypertension (PAH), and interstitial lung disease (ILD) (Fig. 1A). She had no history of atopy or food allergies and used an albuterol inhaler as needed. PAH had been diagnosed seven years prior to presentation when she had a reported pulmonary artery (PA) pressure of 80/39 mmHg, mean PA pressure of 56 mmHg, pulmonary capillary wedge pressure (PCWP) of 12 mmHg, and cardiac output by thermodilution of 3.17 L/min. Her pulmonary function tests (PFTs) at that time showed a forced vital capacity (FVC) of 3.06 L (75% predicted) and a diffusing capacity for carbon monoxide (DL_CO_) of 19.61 mL/min/mm Hg (66% predicted). Per her medical records and history, the patient had been treated with dual therapy (tadalafil and macitentan) for PAH and was subsequently able to wean from oxygen supplementation that she had previously required, indicating a therapeutic response. Her ILD was considered mild, and she did not require supplemental oxygen at rest or with exertion. She was treated with mycophenolate mofetil for a year after her initial diagnosis, but the patient discontinued this medication about 6 years prior to presentation due to fear of reactivating remote Lyme infection after she had read about chronic Lyme disease on the internet. The patient had been stable on her regimen of tadalafil and macitentan until 3 months prior to presentation when she began to experience rapidly progressive dyspnea and new onset hypoxemia. Right heart catheterization (RHC) revealed that her PAH had worsened with a mean PA pressure of 72 mmHg and a new drop in cardiac index (1.7 L/min/m^2^), requiring initiation of subcutaneous treprostinil.

Additional history revealed she had taken oral helminth therapy as an alternative treatment for lcSSc. She ingested *Necator americanus* larvae on 3 separate occasions, 12 weeks apart, with an increasing number of helminths with each dose (3, 5, and 15 larvae). She experienced abdominal pain and diarrhea after the first ingestion. Her respiratory symptoms started 6 weeks after the third and largest dose of helminths.

In the clinic, patient appeared to be in mild respiratory distress with a respiratory rate of 24 breaths per minute, and she required 6–8 L/min of oxygen via nasal cannula to maintain saturations above 90%. She was tachycardic (104 beats/min) with a loud second heart sound, jugular venous distension, and severe lower extremity pitting edema up to her thighs. Auscultation of the chest revealed bibasilar rales. Dermatologic exam was notable for scattered telangiectasias over her face, arms, and hands.

Complete blood count showed leukocytosis (13.5 × 10^3^/uL) with 10% eosinophils (absolute count of 0.98 × 10^3^ cells/uL). Of note, prior blood counts from 2017 had shown normal eosinophil counts (291 × 10^3^ cells/uL). Basic metabolic profile was significant for an elevated creatinine (1.31 mg/dL) but was otherwise normal. Serum brain natriuretic peptide level was elevated at 578 ng/L. IgE was also elevated at 1372.9 IU/mL, and IgG antibodies to *Strongyloides* were detected. Multiple stool analyses were negative for ova and parasites. PFTs at that time revealed an FVC of 2.03 L (49% predicted), a decline of over 1.0 L since her prior testing; complete testing was not possible due to her respiratory symptoms.

High resolution computed tomography (HRCT) of the chest showed worsened subpleural reticulations, traction bronchiectasis, and diffuse ground glass opacities suggesting chronic pulmonary fibrosis in a pattern consistent with non-specific interstitial pneumonia. The patient’s CT scan revealed progressive ILD and showed new diffuse ground glass opacities compared imaging from four years prior (Fig. 1A–D). There were also signs of decompensated right heart failure, including markedly enlarged dilated right heart chambers, pericardial effusion, and flattening of the interventricular septum (Fig. 1E). Though some of the ground glass opacities could have been consistent with pulmonary edema or parenchymal inflammation, additional areas had tract-like abnormalities leading to pleural edge, atypical for either of those entities [2] and not conforming to normal pulmonary vasculature (Fig. 1D).

Immediately after her disclosure of the ingestion of parasites, the patient was treated with albendazole and corticosteroids for presumed Löffler’s syndrome with transient improvement in her dyspnea and hypoxemia. However, due to progressive hypoxemia and signs of decompensated right heart failure on her examination at her outpatient visit, she was admitted to the hospital.

Transthoracic echocardiogram revealed preserved left ventricular function but severely dilated right sided chambers, with a right ventricular systolic pressure estimated at 90 mmHg. Repeat RHC revealed persistently elevated mean PA pressure of 73 mmHg, worsened PCWP of 17 mmHg, and elevated pulmonary vascular resistance of 11.5 Wood units. Due to rapid decompensation and severity of hypoxemia, bronchoscopy was not able to be performed.

Respiratory failure progressed, with the development of refractory hypoxemia and right heart failure that did not respond to aggressive medical therapy in the intensive care unit, including intravenous diuresis and treprostinil as well as inhaled epoprostenol. She was emergently evaluated for lung transplantation but was not offered listing due to her comorbidities, which included severe esophageal dysmotility and renal dysfunction. Her respiratory status continued to deteriorate despite aggressive medical intervetion, and the patient decided to transition to comfort-focused care. She passed away shortly thereafter, and post-mortem examination was declined by her family.

## Discussion and conclusions

After self-administered helminth ingestion, our patient experienced rapidly progressing respiratory failure that may have been multi-factorial, due to worsening pulmonary hypertension and right heart failure, but also with evidence of lung parenchymal abnormalities on imaging consistent with increased inflammation after parasite exposure.

The term “Löffler’s syndrome” refers to pulmonary eosinophilia due to transpulmonary passage of helminth larvae. Several helminths, including Ascaris (*A. lumbricoides, A. suum*), hookworms (*Ancylostoma duodenale, Necator americanus*), and *Strongyloides stercoralis* reach the lungs hematogenously during their normal life cycle [3]. These organisms penetrate from the small alveolar vessels to the alveolar sac and tracheobronchial tree, later swallowed with sputum, thereby enter gastrointestinal tract. Transient radiographic changes in lung fields with associated peripheral blood eosinophilia due to helminthic infection, specifically Ascaris, were first described by Löffler in 1932 [4]. Ascaris is the most common cause of Löffler’s syndrome. Migrating hookworms and Strongyloides are less likely to cause pulmonary eosinophilia and symptoms in immunocompetent hosts.

Patients present with dry cough and substernal discomfort that starts about two weeks after ingestion of eggs or cutaneous penetration of larvae [5]. Fever, dyspnea, wheezing and blood-tinged sputum are also common symptoms. This is usually self-limiting in immunocompetent hosts [6], and symptoms subside in 5–10 days, but can last for more than a month [7, 8]. In rare occasions, when large numbers of larvae are released, heavy hematogenous seeding of helminth larvae can result in lung deposition and prolonged eosinophilic pulmonary inflammation [3]. In addition, Strongyloides have unique life cycle with an ability of continuous autoinfection of the human host [3, 5]. This can result in chronic pneumonitis with pulmonary symptoms and radiographic abnormalities.

Eosinophilia is usually present in association with pulmonary symptoms, and IgE levels may be increased [8]. Eosinophils and Charcot-Leyden crystals can be found in sputum or bronchoalveolar lavage (BAL) fluid [9]. Definitive diagnosis can be made by finding Ascaris, hookworm, or Strongyloides larvae in the respiratory secretions or BAL fluid [1, 5]. Serologic testing for detection of IgG antibodies is available, but specificity is low due to cross-reactivity with filarial species and nematodes [1, 5]. At the time of pulmonary manifestations, stool examinations for ova and larvae are usually negative [5]. Chest imaging can show transient migratory pulmonary opacities [4, 5]. Computed tomography of the chest usually demonstrates peripherally based nodules with a halo of ground glass attenuation. Ground glass opacities are also commonly observed.

Management of Löffler’s syndrome is primarily supportive. Anthelminthic treatment is warranted for suspected or confirmed hookworm or Strongyloides infection regardless of host’s immune status. Albendazole and mebendazole are the first-line antihelminthic drugs for hookworm [8]. If severe pneumonitis is present, systemic glucocorticoids are administered. Before initiating glucocorticoids, Strongyloides infection should be ruled out by examination of sputum and stool to prevent hyper-infection syndrome [1]. Löffler’s syndrome is usually mild and self-limiting disease in immunocompetent hosts [1, 6]. In immunocompromised patients more severe forms of Loffler’s syndrome were reported, mostly due to Strongyloides hyper-infection syndrome [1, 10, 11].

Though ingestion of helminth organisms risks infection, therapeutic ingestion of helminths has been investigated as a potential treatment for autoimmune diseases such as rheumatoid arthritis, psoriasis, multiple sclerosis, Crohn’s diseases, ulcerative colitis, and celiac disease [12, 13]. Although phase I studies suggest that helminth therapy may delay the relapse of Crohn’s disease, it is not an approved therapy for this or any autoimmune disease [14]. Helminth therapy has been associated with reduced disease scores in multiple sclerosis, though significant adverse events were associated with its use in trials [15]. Investigators have hypothesized that administration of helminths could improve autoimmune diseases through immunomodulatory effects, though its use outside of a clinical trial setting is not recommended [15, 16]. Some authors have suggested that helminth extracts may be a safer alternative to ingestion of live organisms, as it would eliminate the risk of infectious complications [15].

In patients with pulmonary symptoms and eosinophilia, complications of parasitic lung disease should remain on the differential diagnosis, and a thorough history including travel and use of alternative medicine treatments is a key for diagnosis. Our patient had ingested *Necator americanus* larvae followed by worsening pulmonary symptoms, new ground glass opacities and tubular parenchymal abnormalities in the setting of known ILD and pulmonary vascular disease secondary to her SSc. The new onset eosinophilia, elevated serum IgE levels, and detection of serum IgG antibodies to Strongyloides point towards parasite-induced inflammation as a cause of her clinical deterioration, which included worsening of her PAH and right heart failure as well as hypoxemia that did not respond to advanced PAH therapies. Though the diagnosis of Löffler’s syndrome in this case cannot be confirmed given the lack of pathology, the temporal relationship between her helminth exposure and rapid clinical deterioration suggests that an inflammatory response to the parasites precipitated her pulmonary decompensation.

To our knowledge, this is the first reported case of rapidly progressive, fatal respiratory failure after self-administration of helminth larvae. It highlights the potential risk of experimental helminth therapy in patients with underlying respiratory disease. This is especially important given the considerable attention to and discussion about helminth therapy on patient-directed forums and social media [17–19].

## Data Availability

Not applicable.

## Abbreviations

BAL: Bronchoalveolar lavage
HRCT: High resolution computed tomography
ILD: Interstitial lung disease
lcSSc: Limited cutaneous systemic sclerosis
PA: Pulmonary artery
PAH: Pulmonary arterial hypertension
PCWP: Pulmonary capillary wedge pressure
RHC: Right heart catheterization
SSc: Systemic sclerosis

## References

[1] Davidson RA (1992). Infection due to *Strongyloides stercoralis* in patients with pulmonary disease. South Med J.

[2] Jeong YJ, Kim KI, Seo IJ, Lee CH, Lee KN, Kim KN (2007). Eosinophilic lung diseases: a clinical, radiologic, and pathologic overview. Radiographics.

[3] Craig JM, Scott AL (2014). Helminths in the lungs. Parasite Immunol.

[4] Loffler W (1956). Transient lung infiltrations with blood eosinophilia. Int Arch Allergy Appl Immunol.

[5] Kunst H, Mack D, Kon OM, Banerjee AK, Chiodini P, Grant A (2011). Parasitic infections of the lung: a guide for the respiratory physician. Thorax.

[6] Mokhlesi B, Shulzhenko O, Garimella PS, Kuma L, Monti C (2004). Pulmonary strongyloidiasis: the varied clinical presentations. Clin Pulm Med.

[7] Hotez PJ, Brooker S, Bethony JM, Bottazzi ME, Loukas A, Xiao S (2004). Hookworm infection. N Engl J Med.

[8] Brooker S, Bethony J, Hotez PJ (2004). Human hookworm infection in the 21st century. Adv Parasitol.

[9] Weissler JC (2017). Eosinophilic lung disease. Am J Med Sci.

[10] Al Hadidi M, Shaaban H, Jumean KH, Peralta P (2018). Loeffler's syndrome secondary to hyperinfection by *Strongyloides stercoralis* associated with methotrexate in a patient with rheumatoid arthritis. J Glob Infect Dis.

[11] Keiser PB, Nutman TB (2004). Strongyloides stercoralis in the immunocompromised population. Clin Microbiol Rev.

[12] Smallwood TB, Giacomin PR, Loukas A, Mulvenna JP, Clark RJ, Miles JJ (2017). Helminth immunomodulation in autoimmune disease. Front Immunol.

[13] Helmby H (2015). Human helminth therapy to treat inflammatory disorders—where do we stand?. BMC Immunol.

[14] Sipahi AM, Baptista DM (2017). Helminths as an alternative therapy for intestinal diseases. World J Gastroenterol.

[15] Correale J (2014). Helminth/parasite treatment of multiple sclerosis. Curr Treat Options Neurol.

[16] Maizels RM, McSorley HJ (2016). Regulation of the host immune system by helminth parasites. J Allergy Clin Immunol.

[17] Parker W. They might sound gross, but intestinal worms can actually be good for you. 2015. https://theconversation.com/they-might-sound-gross-but-intestinal-worms-can-actually-be-good-for-you-49868.

[18] Helminthic therapy personal stories. 2016. https://helminthictherapywiki.org/wiki/index.php/Helminthic_therapy_personal_stories.

[19] Facebook. Helminthic Therapy Support. 2012. https://www.facebook.com/groups/htsupport/discussion/preview.

